# The therapeutic and diagnostic potential of regulatory noncoding RNAs in medulloblastoma

**DOI:** 10.1093/noajnl/vdz023

**Published:** 2019-09-06

**Authors:** Piyush Joshi, Keisuke Katsushima, Rui Zhou, Avner Meoded, Stacie Stapleton, George Jallo, Eric Raabe, Charles G Eberhart, Ranjan J Perera

**Affiliations:** 1 Department of Oncology, Sidney Kimmel Comprehensive Cancer Center, School of Medicine, Johns Hopkins University, Baltimore, Maryland; 2 Cancer and Blood Disorders Institute, Johns Hopkins All Children’s Hospital, St. Petersburg, Florida; 3 Pediatric Neuroradiology, Johns Hopkins All Children’s Hospital, St. Petersburg, Florida; 4 Institute Brain Protection Sciences, Johns Hopkins All Children’s Hospital, St. Petersburg, Florida; 5 Department of Pathology, Johns Hopkins University School of Medicine, Baltimore, Maryland; 6 Sanford Burnham Prebys Medical Discovery Institute, NCI-Designated Cancer Center, La Jolla, California

**Keywords:** circRNAs, diagnostics therapeutics, lncRNAs, Medulloblastoma, microRNAs

## Abstract

Medulloblastoma, a central nervous system tumor that predominantly affects children, always requires aggressive therapy. Nevertheless, it frequently recurs as resistant disease and is associated with high morbidity and mortality. While recent efforts to subclassify medulloblastoma based on molecular features have advanced our basic understanding of medulloblastoma pathogenesis, optimal targets to increase therapeutic efficacy and reduce side effects remain largely undefined. Noncoding RNAs (ncRNAs) with known regulatory roles, particularly long noncoding RNAs (lncRNAs) and microRNAs (miRNAs), are now known to participate in medulloblastoma biology, although their functional significance remains obscure in many cases. Here we review the literature on regulatory ncRNAs in medulloblastoma. In providing a comprehensive overview of ncRNA studies, we highlight how different lncRNAs and miRNAs have oncogenic or tumor suppressive roles in medulloblastoma. These ncRNAs possess subgroup specificity that can be exploited to personalize therapy by acting as theranostic targets. Several of the already identified ncRNAs appear specific to medulloblastoma stem cells, the most difficult-to-treat component of the tumor that drives metastasis and acquired resistance, thereby providing opportunities for therapy in relapsing, disseminating, and therapy-resistant disease. Delivering ncRNAs to tumors remains challenging, but this limitation is gradually being overcome through the use of advanced technologies such as nanotechnology and rational biomaterial design.

Medulloblastoma (MB) is the most common malignant pediatric brain tumor,^1–3^ representing 9.2% of all pediatric brain tumor cases but only 1% of adult cases.^1,4^ MBs arise in the cerebellum and are highly malignant; they commonly metastasize to other parts of the brain and spinal cord and, rarely, to extraneural sites.^5,6^ Transcriptional programs in MBs mimic developmental cerebellar lineages, highlighting their embryonic origin.^7^ The clinical management of MB depends on several factors including molecular and histopathological tumor subgroup, stage, extent of resection and location, and overall patient health. Treatment strategies are aggressive, consisting of a mixture of surgical resection, radiotherapy, chemotherapy, and stem cell/bone marrow transplantation. Despite advances in diagnosis and treatment, MB remains deadly in 35–40% of cases, and those that do survive often suffer long-term side effects including organ dysfunction, neurocognitive impairment, endocrine disabilities, and secondary tumors.^8–11^

Therefore, more effective and less toxic therapies are urgently required to improve clinical outcomes and quality of life for MB patients. In an example of the value of the molecular characterization of cancer, relatively recent molecular classification efforts have refined the clinical and pathological classification of MBs into four clinical/molecular subgroups with distinct driver mutations, cells of origin, and prognoses.^3^ These subgroups provide targets for personalized therapy, some of which are now being tested clinically, but as with many targeted approaches acquired resistance is common, and some subgroups harbor relatively few somatic mutations.^12^ Therefore, the assessment of parameters beyond coding DNA is likely to prove useful not only in terms of understanding the basic biology of MB, but also in expanding the repertoire of molecular targets for refined subclassification, biomarker development, and precision medicine. With this in mind, here we review recent advances in the biology of noncoding RNAs, the nontranslated but functionally active portion of the genome now known to participate in tumorigenesis, in the context of MB. In doing so, we explore how these molecules show particular potential as therapeutics targets.

## The Molecular Classification of Medulloblastoma: A Clinical and Scientific Success Story

MB was traditionally classified histologically into three major types: classic, nodular/desmoplastic (ND), and large cell/anaplastic (LCA), which had prognostic significance but also issues with specificity and reproducibility.^11^ However, and in a valuable illustration of the benefit of molecular characterization of cancer, whole genome, transcriptome, and epigenome analyses have identified significant, clinically relevant molecular heterogeneity between MBs in patient subsets. Since 2016, and in a major restructuring of the classification to include genetically defined entities, the World Health Organization (WHO) Classification of Central Nervous System Tumors divided MB into following major molecular subgroups:^3^ wingless-type (WNT)-activated MB (10%; children and adults, associated with very good prognosis); sonic hedgehog (SHH)-activated MB (30%; intermediate prognosis, infants and adults), further characterized as *TP53* mutant or *TP53* wild-type; and two provisional non-WNT/SHH subgroups: group 3 MB (25%; poor prognosis, infants, and children) and group 4 MB (35%; intermediate prognosis, children, and adults) (reviewed in refs. ^12^ and ^13^). Given that these molecular subgroups have unique clinical and demographic characteristics and prognoses^12–14^ that outperform traditional histopathological classification or clinical staging,^4^ diagnosis is now optimally made using a modular and integrated approach that combines histological and molecular features.

The molecular diagnosis of MB has made it easier to differentiate tumor subgroups, with very reliable diagnostic markers available for WNT and SHH MBs, but group 3 and group 4 tumors are more difficult to define. WNT and SHH MBs generally contain mutations activating these pathways and, aside from rare *TP53*-mutant SHH tumors, are less aggressive than group 3 and 4 tumors.^15^ Biomarkers for WNT MBs include immunohistochemical evidence of YAP1 and nuclear β-catenin, monosomy 6, as well as identification of activating pathway mutations in β-catenin (*CCNTB1*) by sequencing.^16,17^ SHH MBs are generally identified by *GAB1* and *YAP1* co-expression^18,19^ and germline or somatic mutations in *PTCH1* or *SUFU* in children as well as recurrent somatic mutations in *PTCH1*, *SMO*, and the *TERT* promoter in adults.^20,21^ While transcriptional or methylation profiling can also be used to distinguish the four subgroups, this is currently not an accepted clinical assay at most institutions.^17,19,22–26^

The genetic basis of group 3 and group 4 tumors is much less well defined, so their molecular diagnosis has remained challenging, and perhaps unsurprisingly ongoing transcriptomic and genome-wide methylation studies are revealing even greater heterogeneity in these subgroups.^27^ There are currently no reliable immunohistochemical markers for group 3 tumors, although NPR3 positivity has been suggested as a potential biomarker^25^ and MYC levels are also higher in the most clinically aggressive subset of this group. Northcott et al.^28^ recently characterized somatic copy number aberrations (SCNAs) in 1,087 unique medulloblastomas and found tandem duplication of *SNCAIP*, a gene normally associated with Parkinson’s disease, as focal copy number gain exquisitely restricted to group 4 tumors. Recurrent translocations of *PVT1*, including *PVT1-MYC* and *PVT1-NDRG1*, arising via chromothripsis (large-scale genomic rearrangements occurring in a single event in confined genomic regions in one or a few chromosomes) were restricted to group 3 MBs. The presence of numerous targetable SCNAs, including recurrent events targeting TGF-β signaling in group 3 MBs and NF-κB signaling in group 4 MBs, suggests that diagnostic biomarkers for group 3 and 4 tumors will be forthcoming.

## Potential Role of Regulatory Noncoding RNAs in Medulloblastoma Diagnosis and Treatment

While approximately 80% of the human genome is “active,” i.e., transcribed, only about 2% of the genome is protein coding.^29,30^ The remaining transcribed but not translated portion is considered noncoding RNA (ncRNA), the most abundant proportion of which comprises housekeeping ribosomal RNAs (rRNAs) and transfer RNAs (tRNAs).^29,30^ Other nonprotein-coding transcripts were initially regarded and discarded as functionless noise, but ncRNAs are now known to be as important as proteins in regulating cellular function and identity. Based on their size, regulatory noncoding transcripts can be divided into two groups: short noncoding (18–200 nucleotides) and long noncoding RNAs (lncRNAs) (>200 nucleotides). Short noncoding RNAs are further classified into piwi-interacting RNAs (piRNAs), small nuclear RNAs (snRNAs), small nucleolar RNAs (snoRNAs), and microRNAs (miRNAs or miRs). Similarly, long noncoding RNAs can be classified based on location and direction of transcription into long intergenic ncRNAs (lincRNAs), natural antisense transcripts (NATs), enhancer RNAs (eRNAs), and bidirectional transcripts.^31^ Circular RNAs (circRNAs) are a further interesting class of recently discovered ncRNA (reviewed in ref. ^32^; see below) generated from alternative backsplicing of pre-mRNA, with the 3′ and 5′ prime ends of the resulting transcripts covalently binding to produce a circular transcript.

Functionally, regulatory ncRNAs (hereafter referred to as ncRNAs only) regulate transcriptional and post-transcriptional gene expression during development and in disease states. miRNAs regulate gene expression predominantly via post-transcriptional gene silencing through modulating transcript stability.^33^ LncRNAs are functionally diverse and participate in transcriptional silencing (e.g., *XIST*^34^); function as enhancers by regulating three-dimensional chromosomal structure to strengthen interactions between enhancers and promoters (e.g., *LUNAR1*^35^); and sequester miRNAs from their target sites (e.g., TUG1^36^). LncRNAs can also act as hubs for protein–protein and protein–nucleic acid interactions.^37^ CircRNAs are still in their nascent phase, but due their exceptional stability and presence of miRNA binding sites are thought to act as miRNA sponges; i.e., they bind to miRNAs and sequester them.^32,38^ There is now a considerable body of evidence implicating ncRNAs in both health and disease,^39–44^ including in MB. Here we summarize the latest data on ncRNAs in MB and the implications for therapy.

## Mechanistic and Functional Importance of lncRNAs in Medulloblastoma

**Figure 1.**
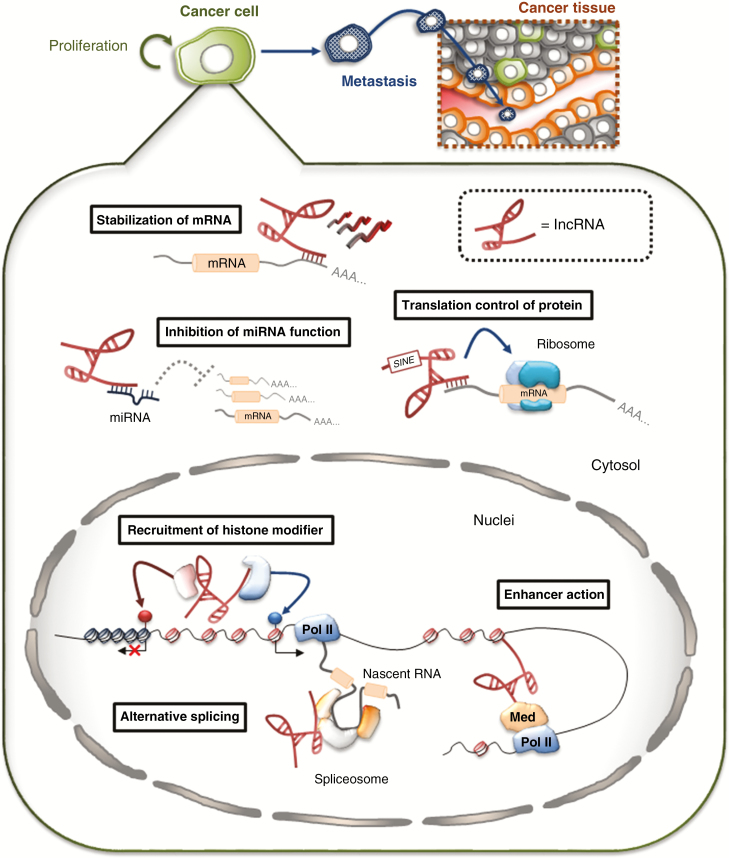
Schematic showing lncRNA functions. lncRNAs are involved in gene regulation through a variety of mechanisms that rely on interactions with multiple molecules. In the cytoplasm, lncRNAs interact with other types of RNA and affect functions including mRNA stability, mRNA translation, or microRNA (miRNA) sponging. In the nucleus, lncRNAs can regulate transcription by recruiting chromatin-modifying complexes by acting as enhancer RNAs (eRNAs). Moreover, they can regulate gene expression by influencing pre-mRNA splicing. Pol II, RNA polymerase II; Med, Mediator complex.

LncRNAs are defined according to length (>200 nt), are transcribed by RNA polymerase II, and commonly originate from intergenic regions. Although the precise roles of the vast majority of the approximately 40,000 known lncRNAs are still uncertain,^45^ at least some of these transcripts are now known to be key regulators of cellular differentiation and proliferation. They are also dysregulated in many types of cancer^46,47^ through their diverse participation in mRNA stability, RNA splicing, chromatin structure, and miRNA‐mediated gene regulation by acting as miRNA sponges^48^ (Figure 1). However, their role in MB is relatively poorly understood, and there are only a few articles on the topic. Here we discuss recently reported mechanistic insights into how lncRNAs regulate gene expression and contribute to MB formation. Table 1 summarizes the lncRNAs implicated in MB together with their molecular partners or genomic targets that mediate MB phenotypes of proliferation, growth suppression, migration, invasion, and metastasis.

**Table 1.** Summary of functions of the main lncRNAs implicated in medulloblastoma

lncRNA	Biological roles in medulloblastoma cell	Molecular functions	Target pathway	Reference
***CCAT1***	Promotion of cell proliferation and metastasis	Unknown	MAPK pathway	^49^
***CRNDE***	Promotion of cell cycle progression	Unknown	Unknown	^50^
***linc-NeD125***	Promotion of cell proliferation, migration and invasion	miRNA sponge (miR-19a-3p, miR-19b-3p and miR-106a-5p)	Unknown	^51^
***LOXL1-AS1***	Promotion of cell proliferation and metastasis	Unknown	PI3K/AKT pathway	^52^
***NKX2-2AS***	Suppression of cell proliferation, migration and invasion	miRNA sponge (miR-103, miR-107 and miR-548m)	SHH pathway	^53^
***PVT1***	Promotion of cell proliferation	Host gene for miRNA (miR-1204, miR-1205, miR-1206, and miR-1207)	Unknown	^28^
***SPRY4-IT1***	Promotion of cell proliferation, migration and invasion	Unknown	Unknown	^54^
***UCA1***	Promotion of cell proliferation and migration	Unknown	Unknown	^55^

## Dysregulated lncRNAs in Medulloblastoma

### linc-NeD125

linc-NeD125, also known as MIR100HG, is significantly overexpressed in group 4 MBs, the largest and least well characterized molecular medulloblastoma subgroup. Mechanistically, linc-NeD125 recruits the miRNA-induced silencing complex (miRISC) and directly binds miR-19a-3p, miR-19b-3p, and miR-106a-5p. Functionally, linc-NeD125 acts as an miRNA sponge that sequesters these three miRNAs and de-represses their targets *CDK6, MYCN, SNCAIP*, and *KDM6A*, which are major driver genes of group 4 MBs. Consistent with the role of linc-NeD125 as an oncogene, ectopic expression of linc-NeD125 promotes medulloblastoma cell proliferation, migration, and invasion in vitro.^51^

### NKX2-2-AS1

NKX2-2-AS1 is involved in SHH-driven MB development. In addition, the SHH pathway transcription factor GLI2 switches on *FOXD1* expression, which subsequently represses transcription of NKX2-2-AS1. Specifically, NKX2-2-AS1 functions as an miRNA sponge to sequester miR-103, miR-107, and miR-548m, thereby maintaining expression of their tumor-suppressive targets BTG2, LATS1, and LATS2. Thus, GLI2/FOXD1-mediated NKX2-2-AS1 downregulation contributes to the pathogenesis of SHH-subgroup MB.^53^

### PVT1

*PVT1* is a noncoding host gene for four miRNAs (miR-1204, miR-1205, miR-1206, and miR-1207) and is amplified together with *MYC* in group 3 MBs. *PVT1* fusion genes are highly recurrent, restricted to group 3 MB, arise through a chromothripsis-like process, and were the first recurrent translocation reported in MB.^28^ The *PVT1* locus is thought to be genomically fragile, as the majority of *MYC*-amplified group 3 MBs harbor *PVT1* fusions. The identified PVT1 fusions involve only PVT1 exon 1 and miR-1204, and only miR-1204 and not the adjacent miR-1205, miR-1206, and miR-1207 are expressed at higher levels in *PVT1-MYC* fusion group 3 MBs compared with nonfusion cases. Inhibition of miR-1204 reduced proliferation of group 3 MB cells at a level comparable to *MYC* knockdown, while an MB cell line with neither *MYC* amplification nor detectable *PVT1-MYC* fusion gene was unaffected by miR-1204 knockdown.^28^ PVT1 stabilizes MYC to promote tumorigenesis, and the *PVT1* locus is often amplified in breast cancer.^56^ However, PVT1 is also frequently disrupted by recurrent translocations or deletions in many cancer types, including MB, suggesting that it may have additional regulatory functions. Of note, a recent study showed that *PVT1* lncRNA expression was not required to inhibit *MYC* transcription; instead, the *PVT1* promoter competed with *MYC* for enhancer binding at the *PVT1* locus, preventing MYC promoter firing and suppressing transcriptional elongation of the *MYC* oncogene and reduced cancer cell growth.^57^ This might indicate a lncRNA-independent tumor suppressive role for the *PVT1* promoter in MB and suggest that regulatory sequences in lncRNA genes may contribute to tumorigenesis.

## MicroRNAs as Theranostic Agents in Human Medulloblastoma

MicroRNAs are a family of endogenous small noncoding RNAs, ~18–25 nucleotide in size, with an evolutionarily conserved structure and a predominant role in posttranscriptional mRNA modifications. Since miRNAs were first discovered in *Caenorhabditis elegans,*^58^ many miRNAs are now known to be crucial cellular regulators in both health and disease.^33^

**Figure 2.**
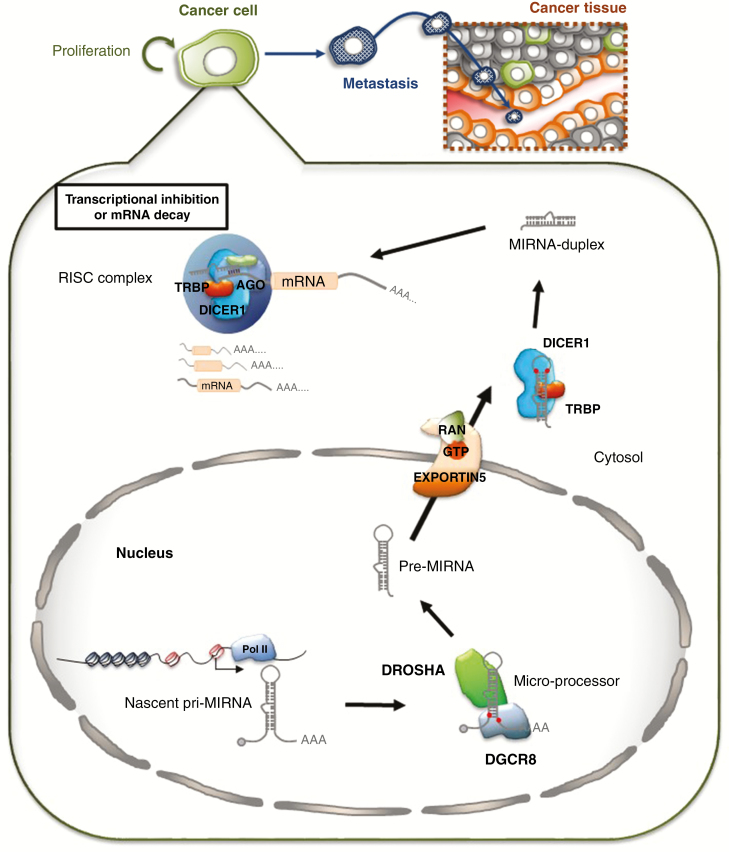
Schematic showing microRNA biogenesis and function. MicroRNAs are synthesized in the nucleus from microRNA encoding genes as longer primary microRNA (pri-miRNA) precursors that are processed by the DROSHA-DGCR8 complex into pre-microRNA (pre-miRNA) molecules. The pre-miRNA transcript is exported out of the nucleus to the cytoplasm where it undergoes a second round of cleavage by the DICER1 complex to form miRNA-duplexes containing the mature miRNA strand. The mature miRNA complexes with the AGO-containing RISC complex and targets mRNA transcripts to inhibit protein production by targeted mRNA decay or posttranscriptional translational inhibition to regulate physiological processes.

miRNAs are synthesized in the nucleus as parent primary-microRNA (pri-miRNA) transcripts, usually by RNA polymerase II^59^ and in certain instances by RNA polymerase III,^60^ with 5′ and 3′ modifications of a normal mRNA^61^ (Figure 2). Due to the characteristic stem-loop structure of the pri-miRNAs, they are recognized by the miRNA processing machinery composed of DGCR8 and the type III RNase DROSHA to be converted into an approximately 85 nucleotide stem-loop structure called precursor miRNA (pre-miRNA).^62^ The pre-miRNA is then transported from the nucleus to the cytoplasm where the final processing step by the RNase III enzyme DICER1 produces mature miRNA in duplex form (miRNA/miRNA*, where * indicates the passenger strand).^62^ The mature single-stranded miRNA is then released from the duplex and incorporated into functional AGO-containing RNA-induced silencing complex (RISC), which guides the complex to target mRNA(s).

Dysregulated miRNA expression is common in human cancer and is crucial for its progression. Common causes of altered miRNA expression in tumors include copy number changes due to amplification, duplication, or deletion, often involving an entire miRNA cluster. In other cases, miRNA dysregulation can be attributed to changes in expression of upstream transcriptional regulators or signaling pathways regulating their expression. For example, frequently mutated cancer genes including *TP53*^63^ or *MYC*^64^ affect a number of miRNAs, resulting in their dysregulation. Functionally, miRNAs are predominantly known to regulate posttranscriptional gene regulation by suppressing gene expression via targeted degradation of RISC-bound transcripts and in some cases via posttranscriptional modifications.^33,62^ miRNAs can act as tumor suppressors or oncogenes depending upon their downstream targets and the cellular context.

### MicroRNAs in Medulloblastoma

As in other tumors, miRNAs are the most studied ncRNA in MB. An early study examining the role of miRNAs in MB revealed overexpression of miR18A, 19A, 21, and 25 in 34 MBs.^65^ Since then, numerous transcriptome-wide comparisons of MBs and control cerebellum tissue have widened the number of candidate miRNAs that might play important roles in MB development.^66–70^ Some of these profiling studies have also associated specific miRNAs with MB subgroups, suggesting a group-specific role in some cases. Several miRNAs including miR-10B, miR-193A, miR-224–452 cluster, miR-182-183-96 cluster, miR-148A, miR-23B, and miR-365 were specifically enriched in the WNT-MB subgroup.^67,69^,^71^ Similarly, overexpression of miRNAs in the miR-17–92 cluster was frequently associated with SHH-MBs and was shown to be oncogenic.^72^,^73^ Gershanov et al. found that 12 miRNAs including miR-181A, 135B, and 660 were overexpressed in group 4 MBs compared with other MBs.^74^ Furthermore, a recent transcriptomic study of the miRNAome of SHH MB cancer stem cells (CSCs) revealed differential up- and downregulation of a number of miRNAs compared with normal neural stem cells including higher expression of miR-20a-5p and miR-193a-5p and lower expression of miR-222-5p, miR-34a-5p, miR-345-5p, miR-210-5p, and miR-200a-3p; some differentially expressed miRNAs in MB CSCs were the same as those dysregulated in primary SHH MB (let-7a, miR-100, miR-132, miR-135a, miR-135b, miR-150, and miR-203).^75^

These studies suggest that miRNAs might have subgroup-specific functions in MBs. We briefly review some of the miRNAs implicated in MB progression, whether they act as oncogenes or tumor suppressor genes, and the associated in vitro/in vivo evidence (Table 2).

**Table 2.** MicroRNAs in medulloblastoma

	miRNA	Expression in MBs	Interactions/Functions	Group specificity	Reference
Oncogenic	miR-21	Upregulated	PDCD4; promotes metastasis		^76^
	miR-17~92 cluster		SHH, MYCN; promotes proliferation	SHH	^73,77^
	miR-183~96~182 cluster		SHH, AKT1/2; promotes proliferation and metastasis		^78^
	miR-30b/d		NA	Group 3	^79^
	miR-10b		BCL2; inhibits apoptosis		^80^
	miR-367		RYR3, ITGAV, RAB23; promotes proliferation of cancer stem cell		^81^
	miR-106b		PTEN; proliferation		^82^
Tumor Suppressor	miR-193		WNT signaling; inhibits cell proliferation	WNT	^67^
	miR-224				^67^
	miR-124	Downregulated	CDK6, SCL16A1; inhibits cell proliferation		^83,84^
	miR-199b		HES1, CD15; inhibits cell proliferation		^85,86^
	miR-125b		SHH signaling, LIFRα; inhibits cell proliferation		^72,85,87^
	miR-324		SMO/GLI1/SHH signaling; inhibits cell proliferation		^72^
	miR-326		SMO/GLI1/SHH signaling; inhibits cell proliferation and self-renewal		^72,88^
	miR-9		t-TrkC; promotes cell cycle arrest and apoptosis		^65^
	miR-125a				^65^
	miR-128a		BMI-1; promotes senescence		^89^
	miR-218		CDK6, RICTOR, CTSB; promotes cell differentiation		^90,91^
	miR-34a		NOTCH signaling, MYCN, SIRT1, MAGE-A; impairs self-renewal and cell proliferation, promotes apoptosis, sensitizes MB cells to chemotherapy		^92–94^
	miR-31		MCM2 regulation; reduces proliferation		^95^
	miR-192		DHFR, CD47; inhibits metastasis		^96^
	miR-135a		ARHGEF6; reduces proliferation		^97^
	miR-494		MYC/p38 MAPK; reduces proliferation, migration, invasion and increases apoptosis		^98^
	miR-466f-3p		Vegf/Nrp2; epithelial to mesenchymal transition		^99^
	miR-221-3p		EIF5A2; cell proliferation, cell cycle and apoptosis		^100^

### Oncogenic miRNAs

Cell cycle and apoptosis pathways are one of the main targets of oncogenic miRNAs in MB. The miRNAs belonging to the miR-17–92 cluster, namely, miR-17, miR-18A, miR-19A/B, miR-20A, and miR-92A, are frequently upregulated in MB samples.^22,73^ The encoding locus was found to be amplified in 6% of MB samples, predominantly in SHH MBs.^73^ In addition, miR17-92 cluster is proposed to be regulated by *MYCN*, a gene frequently amplified in SHH MBs, and overexpression of this cluster promoted cell proliferation even in the absence of SHH signaling.^73^ Further, loss of function studies using locked nucleic acid-mediated knockdown reduced cell growth,^77^ and conditional deletion prevented tumor development in an SHH MB mouse model,^101^ highlighting the oncogenic role of miRNA-17-92 cluster in SHH-dependent tumor growth. Another pro-proliferative miRNA, miR-10b, was found to be upregulated in *ERRB2* (HER2)-overexpressing MBs and in SHH MB and group 3 MB cell lines.^65,80^ miR-10b expression was found to be positively correlated with that of the anti-apoptotic gene *BCL2*, possibly in a positive feedback loop as knockdown of one downregulated the expression of the other.^80^ The authors suggested that miR-10b is important for upregulation/maintenance of *BCL2* expression, thereby promoting proliferation and inhibiting apoptosis.

Metastasis is a poor prognostic event in MBs. miR-21, upregulated in MB compared with normal cerebellum, was shown to promote migration and metastasis^22,76^ by targeting the metastasis suppressor gene *PDCD4*. miR-21 knockdown upregulated *PDCD4*, E-cadherin, and *TIMP2* and consequently downregulated *MAP4K1* and *JNK*. Another metastasis-associated miRNA cluster, miR183-96-182, was also found to frequently co-occur with *MYC* amplifications.^22,102^ Knockdown of these miRNAs individually or together upregulated an apoptosis-associated gene signature and reduced cell viability by impairing DNA repair.^78^ In addition, the cluster genes were shown to have a pro-metastatic role, possibly by regulating divergent AKT1/2 signaling.^78,102^ In a mouse SHH-MB model,^103^ cluster upregulation was frequently associated with *Pten* loss and the cluster genes acted downstream of SHH signaling to promote proliferation.

### Tumor Suppressor miRNAs

Tumor suppressor miRNAs in MB are generally downregulated in response to oncogenic transformations. Some of these miRNAs are involved in promoting neuronal differentiation during normal development. For example, miR-124, a brain-enriched miRNA that usually regulates neurogenesis and neuronal differentiation, is often downregulated in MB.^83,104,105^ In normal brain, miR-124 regulates cell cycle progression by modulating *CDK6*, a well-known marker of high-risk MB,^84,106^ and miR-124 inhibited proliferation in vitro and in vivo upon overexpression in MB cells.^105^ miR-124 has also been suggested to regulate energy metabolism (glycolysis) by targeting the solute carrier SCL16A1.^83^ Similarly, miR-218, another differentiated neuron-enriched miRNA,^107^ was found to be downregulated in MB samples,^90^ particularly in SHH and group 3 patients. In vitro experiments showed that miR-218 overexpression reduced expression of neural stem cell marker *NANOG* and increased expression of neuronal differentiation marker *MAP2*, thereby inhibiting proliferation, clonogenicity, and invasion of SHH MB cells. The study also identified *RICTOR*, *CTSB*, and *CDK6* as miR-218 targets, further implicating the CDK6-miR-218 network in MB proliferation. Furthermore, miR-125b, a member of the let-7 family and a negative regulator of SMO (smoothened),^72^ has been shown to promote neuronal differentiation^85^ and was inversely correlated with *MYC* expression in SHH-MB cells. miR-125b was shown to target leukemia inhibitory factor repressor alpha (LIFRα), which promotes proliferation in vitro and increases tumor volume in vivo.^87^

Tumor suppressor miRNAs are often involved in negatively regulating cell cycle progression and cancer cell stemness. miR-326, a negative regulator of SMO, was found to be downregulated in SHH-MBs, with lower expression associated with high-risk patients.^65,88^ The SHH/GLI-miR-326 network was in particular active in CSCs derived from SHH MBs. CSCs exhibited lower expression of miR-326 and its host gene *Arrb1*. Upon re-induction in SHH-MB CSCs, miR-326 inhibited SHH/GLI signaling at the receptor and transcription factor levels and impaired the self-renewal and proliferative capacity of the cells.^88^ miR-192 was found also found to be downregulated in MB patients, with its overexpression inversely correlated with tumor seeding in cerebrospinal fluid, reduced proliferation and anchoring capability of MB cell lines, and it also directly downregulated the expression of target genes *DHFR, ITGAV, ITGB1/3*, and *CD47*.^96^ Furthermore, xenografted MB cells overexpressing miR-192 in nude mice exhibited reduced leptomeningeal seeding, suggesting that miR-192 is a potential metastasis suppressor.^96^

Notch signaling plays an important role in cell fate decisions in various contexts including neurogenesis and is a frequent direct and indirect target of tumor suppressor miRNAs in MBs. miR-199b, a good prognostic marker, was inversely related to metastases in patients.^85^ In vitro, miR-199b regulated Notch signaling by targeting *HES1*, which in turn inhibited miR-199b expression.^85,86^ miR-199b overexpression in cell lines also decreased the CSC population as judged by reduced expression of CD15 (a direct miR-199B target) and CD133 and inhibited tumor growth in vivo. Another miRNA induced by p53 signaling, miR-34a, targeted Notch ligand Delta-Like 1 (DLL1) in vitro and inhibited CD15^+^/CD133^+^ CSC proliferation in vitro and in vivo while promoting neural differentiation.^92^ miR-34a overexpression also inhibited AKT and STAT3 signaling and was suggested to interact with additional MB pathways such as MYCN, BCL2, and SIRT1.^93^ miR-34A induced apoptosis and cell cycle arrest by directly targeting the oncogene *MAGE*,^94^ the downregulation of the latter inducing p53 signaling to further induce miR-34a to provide positive feedback. In a study specifically examining the CSC compartment in MB, miR-466f-3p was upregulated in CSCs isolated from SHH-MBs derived from *Ptch1* heterozygous mice. In this case, miR-466f-3p suppressed a mesenchymal phenotype via downregulation of *Vegfa* and *Nrp2.*^99^ This result might be of particular significance, since CSCs represent a subset of MB cells that not only promote cancer phenotypes but also participate in chemoresistance.^108^

### Therapeutic Potential of miRNAs in Medulloblastoma

Of all the regulatory ncRNAs, having been the first to be discovered, miRNAs are the closest to reaching clinical applicability. miRNAs are, therefore, important therapeutic targets in many different types of cancer, with several candidates being tested in clinical trials.^109–112^ miRNAs have yet to take a lead in MB therapeutics, but several of the above described miRNAs represent potential therapeutic targets due to their oncogenic or tumor suppressive roles. Here we focus on the therapeutic potential of miRNAs that have shown some promise in in vivo functional validation studies.

miR-34a-dependent downregulation of MAGE-A was shown to sensitize cells to chemotherapeutics such as mitomycin and cisplatin.^94^ Re-introduction of miR-34a in MB cell lines via adenovirus particles inhibited tumor growth in vivo without exhibiting any toxicity.^92^ The tumor suppressor effect of miR-34a was shown in part to be due to regulating Notch signaling crucial for CSC proliferation. miR-199b also targets Notch signaling in CSCs by targeting *HES1*, with similar tumor suppressive results in vivo.^85^ The negative correlation between miR-199b and metastasis and prognosis suggests that miR-199b-based therapy could be used to improve survival in high-risk patients. The pro-proliferation gene *CDK6* is often overexpressed in MB patients and is associated with an unfavorable prognosis, and *CDK6* upregulation could be in part due to downregulation of miRNAs targeting *CDK6* such as miR-124. Sibler et al. showed that reintroduction of miR-124 reduced tumor growth in vivo, suggesting therapeutic potential in a subset of MBs overexpressing CDK6.^105^ MCM2, as a component of the multiprotein MCM2-7 complex, is crucial for DNA replication, transcription, and RNA splicing and is a frequently described oncogene.^113^ miR-31 targeted and downregulated MCM2 in MB cell lines and reduced tumor growth in vivo,^95^ and miR-192 was found to be upregulated in metastatic MB compared with nonmetastatic MB,^96^ inhibiting cellular proliferation and tumor dissemination by targeting integrins.

In the case of oncogenic miRNAs, the miR-17-92 cluster was induced by SHH signaling and *MYCN* and promoted tumor development in vitro and in vivo. Complete knockout of the cluster in SHH-MB mice reduced tumor formation. In addition, locked nucleic acid (LNA)-mediated knockdown of these miRNAs individually or together reduced cell proliferation in vitro and in vivo. Furthermore, intravenous injection of LNAs targeting the miR-17-92 cluster inhibited tumor growth and promoted survival in SHH-MB mice, so may represent a potential therapeutic strategy.^77^

One notable feature of miRNAs in medulloblastoma is their CSC specificity. CSCs show unlimited self-renewal and differentiation capacity, driving cancer progression not only through the generation of functionally diverse progeny but also by being intimately linked to the processes driving dissemination and metastasis, especially epithelial to mesenchymal transition (EMT).^108^ Furthermore, as shown in breast cancer and other solid organ tumors, CSCs are often intrinsically resistant to chemoradiotherapy so contribute to posttreatment relapse: this is also true in MB, and there is recent evidence that the frequent relapses and leptomeningeal dissemination seen in MB patients are caused by therapy-resistant CSCs.^108^ Targeting the CSC compartment is therefore of the utmost importance to fully eradicate the disease. A recent transcriptomic study of the SHH-MB CSC miRNome (compared to background neural stem cells) revealed dysregulation of several KEGG pathways including pathways in cancer, the PI3K-AKT pathway, and the protein processing in endoplasmic reticulum pathway.^75^ In line with these molecular data, most existing studies in MB focusing on eradicating CSCs have focused on specific signaling pathways active in CSCs such as Notch (γ-secretase inhibitors), PI3K/AKT, and STAT3 (celecoxib), with strong preclinical data in mice. For instance, the highly specific PI3K inhibitor GDC-0941 reduced CD133^+^ stem-like MB cell numbers and their clonogenicity and also delayed the growth of highly aggressive group 3 MBs in a xenograft model,^114^ while celecoxib improved the chemoradiosensitivity of CD133^+^ MB xenografts.^115^ Given that miRNAs act upstream of these pathways, they represent attractive candidate targets against the most difficult to eradicate component of the disease and might have particular value in recurrent disease that has failed conventional therapy.

## Other Regulatory Noncoding RNAs in Medulloblastoma

### Enhancer RNAs

Enhancer RNA (eRNA) is a newly identified RNA class that functions as a transcriptional regulator by facilitating high-dimensional DNA structures such that both cis and trans gene interactions link enhancer and super-enhancer DNA sites to transcriptional start sites.^116–118^ eRNA is most likely involved in target gene regulation and chromosome looping.^118–121^ Although eRNA does not appear to be essential for all enhancers to function, eRNA appears to affect the regulation of active enhancer transcription, thereby promoting gene expression and affecting cell-specific transcriptional regulation.^118,122,123^ eRNA is thought to function by directly interacting complexes with RNA pol II as well as DNA- and RNA-binding transcription factors. This interaction induces the formation of genomic looping structures such that enhancers interact with promoters, eRNA, mediator, and cohesin to form 3D tertiary DNA structures and efficient RNA transcription.^124^ Although the role of eRNAs in cancer generally and medulloblastoma specifically is not yet well defined, eRNA levels correlate with enhancer activity and eRNA can be used as a genome-wide marker of active enhancer elements.^125^ In addition, recent studies have demonstrated that aberrant regulation of eRNAs transcribed from super-enhancers is often linked to cancer development,^126–128^ suggesting that eRNAs may be important cancer targets. Using H3K27ac and BRD4 chromatin immunoprecipitation followed by sequencing coupled with tissue-matched DNA methylation and transcriptome data, Liu et al. described the active cis-regulatory landscape across 28 primary MBs.^129^ Analysis of differentially regulated enhancers and super-enhancers reinforced intersubgroup heterogeneity and revealed novel, clinically relevant insights into medulloblastoma biology.^129^ eRNA may play an important role in MB phenotypic changes and responses to tumor microenvironmental changes.

### Circular RNA

Recent advances in deep-sequencing technology and computational biology have further expanded the repertoire of regulatory ncRNAs, with *circ*ular *RNA*s (circRNAs) one of the latest additions. Besides their unique configuration, circRNAs are distinct from their canonical linear siblings in that they harbor frequent exon scrambling events. circRNAs are products of rare “head-to-tail” back-splicing events catalyzed by the splicing machinery. This back-splicing reaction is favored when pre-mRNAs adopt a noncanonical configuration by juxtaposing the 3′ end of a downstream exon to the 5′ end of an upstream exon. Back-splicing is subject to regulation by *cis*-regulatory elements and/or *trans*-acting factors. For example, complementary base-pairing by transposon-derived inverted repeats flanking circRNA-generating exons or RNA elements that contain recognition motifs for select RNA-binding proteins (RBPs) can promote the aforementioned noncanonical pre-RNA configurations, thereby facilitating circRNA biogenesis.^130,131^ In addition, limited splicing machinery (upon depletion of select spliceosome components) or an increase in the occurrence of transcription read-through events (upon a reduction in levels of components of the pre-mRNA 3′ end processing machinery) also promote back-splicing.^132^

Originally viewed as incidental by-products of rare “head-to-tail” back-splicing events, circRNAs are now known to be an abundant class of RNAs present in a wide variety of eukaryotes, including nematodes, flies, mice, and humans.^131,133–141^ An increasing number of circRNAs are being functionally characterized. For example, the mouse circRNA *CDR1as/CiRS-7* sequesters miR-7 and affects brain development.^135,138,142^ The circRNA *SRY* plays a key role in male sex determination.^135,138,142^ Furthermore, select intron-containing circRNAs can interact with U1 snRNP and promote host gene transcription.^143^ In addition, circRNAs can function in gene regulation by competing with linear splicing.^144^ Select circRNAs can also give rise to functional polypeptides, thereby expanding proteome complexity.^145,146^ Given that circRNAs are highly enriched in the brain and that select circRNAs have been implicated in cancer,^140,147^ it is possible that circRNAs participate in the physiopathology of MB. In a recent study by Lv et al.,^148^ expression profiling of circRNAs in a small sample of MBs found 33 differentially expressed circRNAs compared with control cerebellum. The study identified three of these 33 circRNAs to be overexpressed in the MB tissue. Specifically, MB-enriched circRNAs circ-SKA3 and circ-DTL promoted proliferation and migration in vitro, possibly by regulating host gene expression. As the field of circRNA is in its nascent phase, future studies will shed light on putative candidates for biomarker and therapeutic end goals.

## Therapeutic Targeting of ncRNAs in Medulloblastoma: Technical Considerations and Limitations

We have described several lncRNAs and miRNAs that show MB subgroup-specific expression and regulating oncogenes and/or tumor suppressor genes,^149^ making them putative diagnostic biomarkers and therapeutic targets in MB. While the evidence level for majority of candidates is still in nascent phase lacking in vivo mouse studies, nevertheless in vitro studies highlight the potential of candidate factors.

Several approaches could be used to target lncRNAs in cancer including MB: posttranscriptional degradation through argonaute- and dicer-dependent cleavage with siRNAs or RNase H-dependent degradation with modified antisense oligonucleotides; regulation of lncRNA expression with CRISPR-Cas9 gene editing or promoter blockade; or inhibition of RNA-protein interactions with small molecule inhibitors or antisense oligonucleotides.^149^ Likewise, similar strategies have been used to target miRNAs: antisense oligonucleotides that anneal to the mature miRNA, often modified (especially with 2’-O- methyl groups) to improve stability, affinity, and specificity; LNA anti-miRNA constructs, which have extremely high affinity to single-stranded RNA; miRNA sponges and miR-masks, transcripts that contain multiple miRNA tandem binding sites (sponges) or single-stranded 2′-O-methyl-modified antisense oligonucleotides complementary to predicted miRNA binding sites to compete with cellular miRNA target sites; or upstream small molecule inhibitors to interfere with their synthesis or processing.^150^ Conversely, miRNAs or lncRNAs can be reintroduced into tumors as mimicking synthetic oligonucleotides that re-introduce tumor suppressor function.^151^

There has been some advances in targeting lncRNAs and miRNAs with nucleic acid-based therapies both in vitro and in vivo; ^152^ however, several challenges stills remains to be addressed. First of all, effective delivery remains an important barrier, particularly in vivo, since (i) nucleic acids require active and protected transport to cross plasma membranes; (ii) cellular nucleases and the innate immune response degrade nucleic acids; and (iii) nucleic acids can become sequestered or degraded in the endosomal compartment.^36^ Additionally, given the brain location of MBs, the blood-brain-barrier (BBB) presents an MB-specific challenge to delivery, not only for small molecules (most of which cannot cross the BBB) but also for nucleic acids, whose high molecular weight, anionic charge, and instability pose additional challenges.^11,153^ Several strategies exist to overcome the BBB including viral carriers (e.g., with adeno-associated virus) or nonvirus methods such as hijacking the BBB transcytosis machinery via transferrin receptors and clathrin and caveolae-mediated endocytosis; direct intrathecal injection; or temporarily disrupting the BBB.^154^

One of the side effects of currently used treatment options is the deterioration/delayed development of cognitive and behavioral potential of a treated child; ^8,9^ particularly with surgical, chemo, and radiation approaches. RNA-based therapeutic approaches has been suggested to confer reduced toxicity due to reduced systematic exposure and incorporation of modifications for that specific purpose.^155–157^ For example, while overexpression of siRNA can saturate normal miRNA machinery that could indeed result in severe toxicity,^158^ designing siRNA as asymmetric DICER substrate could potentially address off-target effects.^159^ Another source of toxicity in RNA-based therapeutic approaches could be the choice of delivery system, as seen in the case of nanoparticles, in that case, conjugate-based approach offers better alternative.^160^

Altogether, as in the case of other cancer, ncRNA offers new therapeutic options for MB treatment. While significant challenges remain to identify specific RNA targets for their efficacy and amenability, we must additionally address the cost-to-benefit balance between treatment and overall life quality posttreatment in selecting new therapeutic approaches.

## Conclusions

Various ncRNAs are now known to participate in MB biology, although the functional significance of many remains uncertain. Nevertheless, different lncRNAs and miRNAs have oncogenic or tumor suppressive roles in MB. By contributing to the observed heterogeneity between subgroups, these ncRNAs possess subgroup specificity that can be exploited to personalize therapy both by acting as biomarkers and as therapeutic targets. Of note, several of the already identified ncRNAs are specific to MB CSCs, the most difficult-to-treat component of the tumor that drives metastasis and acquired resistance, thereby providing opportunities for therapy in relapsing, disseminating, and therapy-resistant disease. Delivering nucleic acids to tumors—and especially central nervous system tumors behind the blood-brain-barrier—remain a technical challenge but one that is gradually being overcome through the use of advanced technologies such as nanotechnology and rational biomaterial design.

## Notes

**Authorship statement**. All authors participated in writing, reviewing, and editing the manuscript.
